# Incidence, risk factors, and outcomes of central venous catheter‐related thromboembolism in breast cancer patients: the CAVECCAS study

**DOI:** 10.1002/cam4.1201

**Published:** 2017-10-04

**Authors:** Philippe Debourdeau, Marc Espié, Sylvie Chevret, Joseph Gligorov, Antoine Elias, Pierre François Dupré, Kristell Desseaux, Issa Kalidi, Stephane Villiers, Sylvie Giachetti, Corinne Frere, Dominique Farge

**Affiliations:** ^1^ Department of Medical Oncology Sainte Catherine Institute Avignon France; ^2^ Breast Cancer Unit Saint Louis Hospital Assistance Publique‐Hôpitaux de Paris Paris France; ^3^ University Paris Diderot Paris France; ^4^ Biostatistic Department and Medical Informatics Saint‐Louis Hospital Assistance Publique‐Hôpitaux de Paris Paris France; ^5^ Medical Oncology Tenon Hospital Assistance Publique‐Hôpitaux de Paris Paris France; ^6^ Francilian Breast Intergroup, APREC IUC‐UPMC Sorbonne University Paris France; ^7^ Department of Vascular Medicine Sainte Musse Hospital Toulon France; ^8^ Department of Gynecology and Surgery CHRU Brest France; ^9^ Department of Biology Saint Louis Hospital Assistance Publique‐Hôpitaux de Paris Paris France; ^10^ Department of Anesthesiology and Reanimation Saint Louis Hospital Assistance Publique‐Hôpitaux de Paris Paris France; ^11^ Department of Haematology Pitié‐Salpêtrière Hospital, Assistance Publique‐Hôpitaux de Paris Paris France; ^12^ Sorbonne Universités UPMC Université Paris 06, UMR_S 1166 Paris France; ^13^ Institute of Cardiometabolism and Nutrition, ICAN Paris France; ^14^ Internal Medicine Unit: Autoimmune and Vascular Diseases, UF 04 Saint‐Louis Hospital, Assistance Publique‐Hôpitaux de Paris, AP‐HP Paris France

**Keywords:** Breast cancer, central venous catheter, chemotherapy, risk factors, venous thromboembolism

## Abstract

Previous epidemiologic studies investigating central venous catheter (CVC)‐related venous thromboembolism (CRT) were conducted in heterogenous cancer populations and data in breast cancer (BC) remain limited. To investigate the Doppler ultrasound (DUS)‐CRT incidence, risk factors and outcomes in BC, we designed a prospective, multicenter cohort of nonmetastatic invasive BC patients undergoing insertion of a CVC for chemotherapy. All patients underwent double‐blind DUS before, 7, 30, and 90 days after CVC insertion and a 6 months clinical follow‐up. Symptomatic DUS‐CRT were treated by anticoagulants. D‐Dimers, thrombin generation, and platelet‐derived microparticles were measured before and 2 days after CVC placement. In DUS‐CRT patients, a nested case–control study analyzed the role of thrombophilia. Among 524 patients, the DUS‐CRT (14 symptomatic, 46 asymptomatic) cumulative probability was 9.6% at 3 months and 11.5% at 6 months (overall incidence rate: 2.18/100 patient‐months). Ten/14 symptomatic DUS‐CRT were detected on double‐blind DUS before the clinical symptoms, and 3/14 had a simultaneous pulmonary embolism. No clinical thrombotic event subsequently occurred in untreated asymptomatic DUS‐CRT. Age >50 years (OR, 1.80; 95% CI, 1.01–3.22), BMI >30 kg/m² (OR, 2.64; 95% CI, 1.46–4.76) and comorbidities (OR, 2.05; 95% CI, 1.18–3.56) were associated with DUS‐CRT. No biomarkers was found to predict DUS‐CRT. In multivariate analysis, BMI >30 kg/m² (OR, 2.66; 95%CI, 1.46–4.84) and lobular carcinoma histology (OR, 2.56; 95%CI, 1.32–4.96) remained the only significant DUS‐CRT risk factors. Thrombophilia did not account for DUS‐CRT. Only clinical parameters identified high risk DUS‐CRT patients who may be considered for thromboprophylaxis.

## Introduction

Breast cancer (BC), accounting for 25% of women cancers, is the most frequent cancer and the leading cause of death in the female population worldwide 1. As compared to their age‐matched individual counterparts without cancer, BC women have a three to fourfold increased risk of venous thromboembolism (VTE) 2, 3, which was notably demonstrated to be an independent prognostic factor for survival and the second cause of death in all cancer patients 4, 5, 6, 7. A large UK registry study 6, 8 recently showed that the VTE risk in BC patients is specifically higher around the diagnosis period and until 3 months later, particularly on (neo)adjuvant chemotherapy (NAC) 6, meanwhile long‐term central venous catheters (CVC) are commonly inserted to facilitate intravenous administration of treatments. While risk factors for VTE in BC women have been extensively investigated 6, data regarding CVC‐related thrombosis (CRT) are scarce and factors promoting CRT in this specific population remain poorly understood. Previous epidemiologic cohort studies were conducted only in heterogeneous cancer patient populations. Verso et al. first reported an overall incidence of 4–5% (0% to 28%) for symptomatic CRT and 30% (27% to 66%) for asymptomatic CRT detected by venography in unselected cancer patients 9. The incidence of CRT further varies widely between studies, due to many differences in CRT definition and diagnostic procedures. Importantly, symptomatic CRT have been found to result in pulmonary embolism (PE) in 10–15% of unselected cancer patients 9, 10. On the other side, the clinical consequences of an asymptomatic CRT diagnosed on various imaging tools still remain uncertain, particularly concerning its spontaneous outcome without anticoagulation treatment and if its early diagnosis may predict the onset of symptomatic CRT 11. In the absence of evidence, anticoagulants are not currently recommended for CRT prophylaxis 12, and the ASCO guidelines on CVC care for cancer patients highlighted the need for additional research in this area 13. We, therefore, designed the prospective multicenter CAVECCAS (Cathéter VEineux Central et CAncer du Sein) study in a highly selected population of nonmetastatic invasive BC patients to investigate the specific incidence, risk factors and outcomes of both asymptomatic and symptomatic CRT in BC patients within the 6 months after CVC insertion for NAC.

## Materials and Methods

### Study design

This multicenter observational cohort study was conducted in nine cancer hospitals between September 2008 and December 2011. All nonmetastatic invasive BC patients were screened. The inclusion criteria were histologically proven BC cancer patients older than 18 years to be treated by adjuvant chemotherapy or NAC necessitating the insertion of a port single central lumen catheter for more than 3 months 14. All CVC were inserted via the internal jugular, axillary or subclavian veins and terminated in the superior vena cava. We excluded patients receiving NAC via peripherally inserted catheter, tunneled catheters without port and femoral CVC, patients treated by a previous chemotherapy and/or hormonal therapy, patients on curative anticoagulant therapy, patients with a platelet count <80G/L, a INR <1.5, a fibrinogen level < 1 g/L, and a creatinine level >175 *μ*mol/L. All eligible consecutive patients were prospectively enrolled after obtaining their written informed consent to participate in the trial. The study was approved by Ethical Committee of Paris (France) and registered on clinical trial.gov (ClinicalTrials.gov Identifier: NCT00714909).

### Data collection

Baseline patients' demographic and clinical characteristics, cancer characteristics, site (jugular or subclavian) and side (right or left) of CVC insertion were recorded as well as the insertion procedure duration and the number of venipunctures (>2). According to Good Clinical Practices Guidelines 15, Doppler‐ultrasonography (DUS) was recommended to guide CVC insertion, which had to be preferably positioned on the right side 10, 14, 16, with CVC distal tip at the superior vena cava and the right atrium junction. In women with right BC, CVC insertion was allowed on the left side.

### Outcome measures

All patients underwent double‐blind DUS before and 7, 30, and 90 days (D) after CVC insertion, including compression, B‐mode imaging with the addition of color and pulsed‐wave Doppler. The following parameters were recorded on both sides on the humeral, axillary, subclavian, internal jugular veins, and when possible on the brachiocephalic and superior cava veins: venous vessels patency, presence or absence of vein compressibility, echogenicity within the vein lumen, characteristics of venous flow, including presence or absence of cardiac pulsatility transmitted, response to respiratory maneuvers. The main outcome measure was the occurrence of either: (a) asymptomatic CRT detected by repeated DUS performed at each investigating site by the same radiologist at day 7 (±2), day 30 (±5) and day 90 (±7) after CVC insertion or (b) symptomatic CRT over the 6 months of patients clinical follow‐up diagnosed by any VTE clinical symptoms of the upper limb, neck, or head (pain, edema, or headache) or PE, and objectively confirmed by DUS, phlebography, angiography, computed tomography, or pulmonary scintigraphy. CRT was defined as the occurrence of a mural thrombus extending from CVC into the lumen 15. For veins accessible to direct insonation, diagnostic criteria of CRT were: noncompressibility, visualization of echogenic intravascular mass and absence of respiratory variation (jugular, axillary or subclavian veins) 17. For veins inaccessible to direct insonation (middle part of the subclavian vein, brachiocephalic vein and superior caval vein) the criterion of monophasic flow to detect occlusive thrombosis was used. All DUS thrombotic events were reviewed by two physicians (DF, PD) unaware of clinical data. When an asymptomatic CRT was diagnosed on DUS as scheduled by systematic examination according to the study protocol, patient and referring physicians were blind to DUS results. CVC dysfunction not related to CRT, such as distal thrombus without mural involvement or a fibrin sleeve around the CVC distal tip, or pinch‐off syndrome were not recorded as an event. According to the study protocol, asymptomatic CRT were not to be treated, and thrombus evolution was assessed upon next DUS examination. Anticoagulant treatment was initiated only at the onset of clinical symptoms for symptomatic CRT 17.

### Blood samples and laboratory analysis

Venous blood samples were collected before and 2 days after CVC insertion. D‐Dimers levels were measured, using an Enzyme Linked Fluorescent Assay (VIDAS^®^ D‐Dimer Exclusion^™^, Biomérieux, Marcy l'Etoile, France) on a Mini Vidas analyzer (Biomérieux). Platelet‐derived MPs (Pd‐MPs) and Pd‐MPs expressing phosphatidyl serine (Pd‐MP/PS+) were measured, using a flow cytometry assay as previously described 18. Thrombin generation was studied, using the Calibrated Automated Thrombogram assay (CAT^®^, Stago, Asnieres, France) according to the manufacturers' instructions with PPP reagent 5 pM^®^ (Thrombinoscope b.v., Maastricht, the Netherlands). Each patient's plasma was studied in duplicate. In a third well, PPP reagent 5 pM^®^ was replaced with the same volume of Thrombin Calibrator^®^ (Thrombinoscope b.v., Netherlands) to correct thrombin generation curves for substrate consumption and inner filter fluorescence effects. The following thrombogram parameters were analyzed: the lag‐time of thrombin generation, the time to reach the peak of thrombin (time to Peak), the thrombin peak (Peak), the endogenous thrombin potential (ETP), which reflects the total amount of thrombin activity. Additional individual heritable or acquired thrombophilia risk factors were analyzed in patients selected for the nested case‐control study. It included antithrombin, protein C and protein S levels measured on a STAR‐R analyzer (Stago) using the STACHROM and STACLOT assays (Stago); search for Lupus anticoagulant performed on a STAR‐R analyzer, using the STACLOT DRVV and the PTT‐LA assays (Stago); dosage of anticardiolipin and anti*β*2GP1 antibodies levels measured with an ELISA method (respectively, Stago, Biorad, Phadia); and search for the G1691A polymorphism in the gene encoding factor V (FVL) and the prothrombin G20210A polymorphism identified, using an allele‐specific restriction‐enzyme analysis as previously described 19, 20.

### Nested case–control study

After completing recruitment and follow‐up, a nested case–control study was performed to analyze additional individual thrombophilia risk factors (Antithrombin, Protein C and Protein S levels, presence of Factor V and Factor II Leiden mutations, presence of antiphospholipid, anticardiolipin and anti*β*2GP1 antibodies). Two controls without CRT were matched for TNM status with each symptomatic or asymptomatic CRT patient from the CAVECCAS cohort.

### Statistical analysis

Statistical analysis was performed on the open‐source software R Version 2.15.2. R foundation, Vienna, Austria Summary statistics are expressed as median and inter‐quartile range [IQR] and (minimum, maximum) for quantitative data and numbers and percentages for categorical data. Continuous variables with a skewed distribution were log‐transformed. Univariate comparisons used the exact Fisher test or the Wilcoxon rank sum test according to the variable type. In the multivariate models, a step‐down selection procedure was used with *P*‐values < 0.10 as the selection criterion. A conditional logistic model was used to study the occurrence of thrombosis based on thrombophilia factors testing.

## Results

### Study population

**Figure 1:**
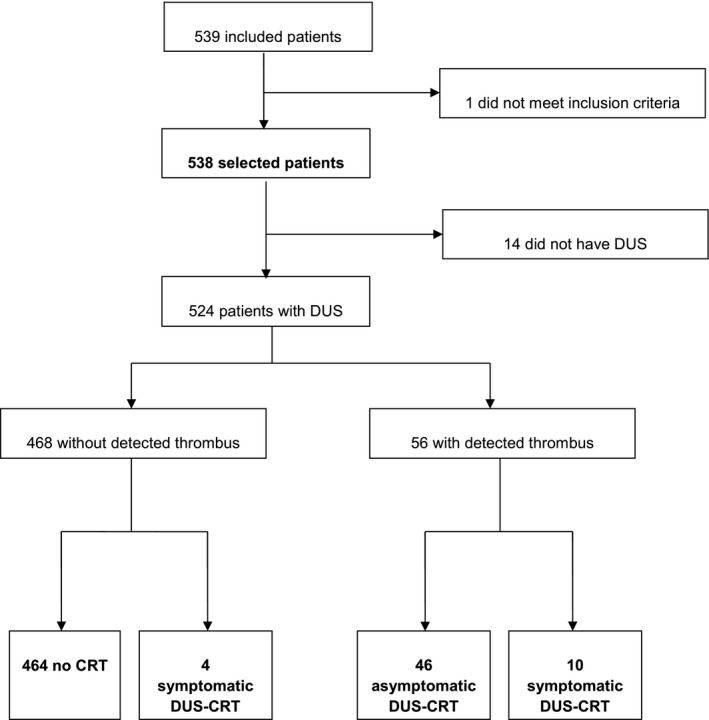
CAVECCAS study flow‐chart.

Here, between September 2008 and December 2011, 539 consecutive patients were enrolled in the study. One patient did not meet all the inclusion criteria, and 14 did not attend the DUS examination. Finally, 524 patients were included in the CAVECCAS study cohort. Flowchart is displayed in Figure 1. Baseline patients' demographic and clinical characteristics, as well as cancer characteristics and treatments are shown in Table 1. At inclusion, patients had a median age of 53 years (interquartile range [IQR], 46–62 years) and 523 were women (99.8%). One hundred forty‐five patients (27.7%) presented one or more comorbidities, including diabetes mellitus, hypertension, liver failure, kidney failure, chronic respiratory failure, or cardiac failure. Twelve patients (2.3%) had a previous VTE history (7 with lower limb DVT, 5 with PE). Other VTE risk factors were obesity with Body Mass Index (BMI) above 30 kg/m² (*n* = 89), lower limb varicose (*n* = 50), previous thoracic venous catheter or traumatism (*n* = 5) or hereditary thrombophilia (*n* = 1). At enrollment, 85.9% of patients (*n* = 450) had an invasive ductal carcinoma, 49.2% (*n* = 230) had node involvement, and 79.5% (*n* = 415) were positive for either estradiol or progesterone receptors. All CVC were single port CVC from various manufacturers brands, namely Braun (272), Perouse (*n* = 113), Bard (*n* = 92), Districath (*n* = 3), Heliosite (*n* = 2), Vygon (*n* = 1), or others (*n* = 41).

**Table 1:** Breast cancer patients characteristics and venous thrombosis risk factors at enrollment

	All (*n* = 524)	No CRT (*n* = 464)	CRT (*n *= 60)	*P*
Breast cancer side, *n* (%)				1.00
Left	271 (52)	240 (52)	31 (51.7)	
Right	251 (48)	222 (48)	29 (48.3)	
Missing	2	2	0	
Histological type, *n* (%)				0.016
Ductal carcinoma	445 (85.0)	400 (86.2)	45 (75.0)	
Lobular carcinoma	64 (12.2)	51 (11.0)	13 (21.7)	
Ductal and lobular carcinoma	5 (0.9)	3 (0.6)	2 (3.3)	
Other	10 (1.9)	10 (2.2)	0	
TNM staging, tumour *n* (%)				0.025
T0	2 (0.5)	0	2 (3.3)	
T1	209 (47.6)	188 (49.6)	21 (35.0)	
T2	190 (43.3)	164 (43.3)	26 (43.3)	
T3	35 (8.0)	33 (8.7)	2 (3.3)	
T4	3 (0.7)	3 (0.8)	0	
Missing	85	85	0	
TNM staging, node (%)				1.00
N0	254 (48.6)	224 (48.5)	30 (50.0)	
N1	207 (39.6)	183 (36.9)	24 (40.0)	
N2	48 (9.2)	43 (9.3)	5 (8.3)	
N3	13 (2.5)	12 (2.6)	1 (1.7)	
Missing	2	2	0	
Node involvement, *n* (%)				0.77
No	223 (49.2)	195 (48.9)	28 (51.9)	
Yes	230 (50.8)	204 (51.1)	26 (48.2)	
Missing	74	68	6	
Steroid hormone receptors, *n* (%)				1.00
No	107 (20.5)	95 (20.6)	12 (20.0)	
Yes	415 (79.5)	367 (79.4)	48 (80.0)	
Missing	2	2	0	
SBR grading, *n* (%)				0.74
1	57 (11.2)	49 (10.8)	8 (13.8)	
2	271 (53.0)	242 (53.4)	29 (50.0)	
3	183 (35.8)	162 (35.8)	21 (36.2)	
Missing	13	11	2	
HER2 status, *n* (%)				0.85
Negative	443 (84.9)	391 (84.6)	52 (86.7)	
Positive	79 (15.1)	71 (15.4)	8 (13.3)	
Missing	2	2	0	
Chemotherapy, *n* (%)				0.25
Anthracycline	116 (22.2)	99 (21.4)	17 (28.3)	
Anthracycline + taxanes	407 (77.8)1	364 (78.6)1	43 (71.7)0	
Missing	1	1	0	
Herceptin	76 (14.6)	69 (14.9)	7 (11.7)	0.70
Previous DVT, *n* (%)				0.57
No	516 (98.7)	458 (98.7)	58 (98.3)	
Yes	7 (1.3)	6 (1.3)	1 (1.7)	
Missing	1	0	0	
Previous PE, *n* (%)				1.00
No	519 (99.1)	459 (98.9)	61 (100)	
Yes	5 (0.9)	5 (1.1)	0	
Obesity (BMI >30 kg/m^2^), *n* (%)				0.044
No	433 (82.9)	389 (84.2)	44 (73.3)	
Yes	89 (17.1)	73 (15.8)	16 (26.7)	
Missing	2	2	0	
Lower limbs varicose, *n* (%)				0.82
No	474 (90.5)	420 (90.5)	54 (90.0)	
Yes	50 (9.5)	44 (9.5)	6 (10.0)	
Previous thoracic venous catheter, *n* (%)				1.00
No	523 (99.8)	463 (99.8)	60 (100)	
Yes	1 (0.2)	1 (0.2)	0	
Known thrombophilia, *n* (%)				1.00
No	522 (99.8)	462 (99.8)	60 (100)	
Yes	1 (0.2)	1 (0.2)	0	
Missing	1	1	0	
Comorbidities ≥1, *n* (%)				0.014
No	379 (72.3)	344 (74.1)	35 (58.3)	
Yes	145 (27.7)	120 (25.9)	25 (41.7)	
Diabetes mellitus, *n* (%)				0.34
No	498 (95.0)	439 (94.6)	59 (98.3)	
Yes	26 (5.0)	25 (5.4)	1 (1.7)	
Hypertension, *n* (%)				0.006
No	395 (75.4)	359 (77.4)	36 (60.0)	
Yes	129 (24.6)	105 (22.6)	24 (40.0)	
Liver failure, *n* (%)				0.19
No	523 (100.0)	463 (100.0)	60 (100.0)	
Yes	0	0	0	
Missing data	1	1	1	
Kidney failure, *n* (%)				1.00
No	524 (100.0)	464 (100.0)	60 (100.0)	
Yes	0	0	0	
Chronic respiratory failure, *n* (%)				1.00
No	516 (98.5)	457 (98.5)	59 (98.3)	
Yes	8 (1.5)	7 (1.5)	1 (1.7)	
Cardiac failure, *n* (%)				1.00
No	520 (99.8)	460 (99.8)	60 (100.0)	
Yes	1 (0.2)	1 (0.2)	0	
Missing data	3	3	0	

CRT, catheter‐related thrombosis; TNM, Tumour, Node, Metastases; SBR, Scarff Bloom et Richardson grading; HER2, human epidermal growth factor receptor 2; DVT, deep vein thrombosis; PE, pulmonary embolism.

### Catheter‐related thrombosis

**Figure 2:**
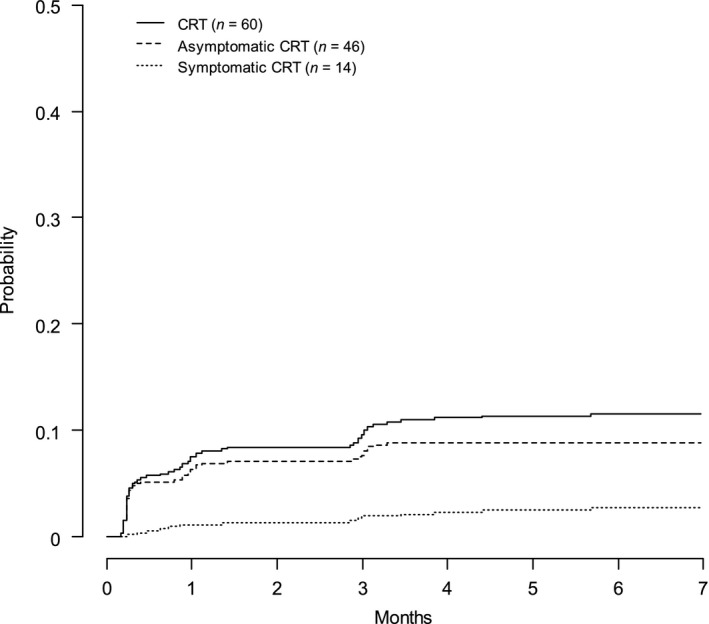
Incidence of catheter‐related thrombosis in the CAVECCAS study.

During the 180 days of clinical follow‐up, 60 patients developed a DUS‐CRT. The final DUS‐CRT incidence rate was 2.18 cases per 100 patient‐months. The cumulative probability of DUS‐CRT was 9.6% after 3 months and 11.5% after 6 months (Fig. 2). DUS‐CRT events were symptomatic in 14 patients (2.7%) and remained asymptomatic in 46 patients (8.8%) up to 6 months of clinical follow‐up. Among the 14 symptomatic CRTs, 10 were detected on DUS before the onset of symptoms (median 6.5 [IQR], 0–17.5 days before the onset of symptoms) and 3 presented simultaneous PE. All symptomatic patients were treated according to the international guidelines 12. In the 46 asymptomatic patients, presence of mural thrombus was detected on study protocol repeated DUS at day 8, 30 and 90 after CVC insertion in, respectively, 27 (46%), 10 (22%), and 9 (19%) patients, and thrombus resolved on the next study protocol DUS in 30 patients. Due to protocol deviation, 5 asymptomatic CRTs were treated by anticoagulation. In the 41 patients with asymptomatic CRT who remained untreated, no symptomatic CRT or PE subsequently occurred.

### Biological parameters

Among the 524 selected patients, 501 (95.6%) had coagulation blood samples before and after catheter insertion (Table 2). The median D‐Dimers value was statistically higher after (586 [366; 842] ng·mL^−1^) than before CVC insertion (454 [294; 757] ng·mL^−1^; *P* < 0.0001). No significant increase in thrombin generation peak height (257 [194; 307] vs. 253 [201; 308] nmol) nor in endogenous thrombin potential (1304 [1063; 1652] vs. 1322 [1052; 1582] nmol·min) were observed. Both Pd‐MPs (982 [518; 2147] vs. 759 [417; 1373] mL^−1^; *P* < 0.0001) and Pd‐MPs/PS+ (778 [409; 1851] vs. 730 [381; 1412] mL^−1^; *P* = 0.021) levels were significantly lower after CVC insertion.

**Table 2:** Coagulation tests before and 2 days after central venous catheter insertion in all CAVECCAS patients

Biomarkers	Before catheter insertion Median (interquartile range)		After catheter insertion Median (interquartile range)		*P*‐value (sign rank Wilcoxon test)
D‐Dimers (ng·mL^−1^)	*n* = 490	454 (294.2–756.5)	*n* = 465	586 (366–842)	<0.0001
Thrombin generation test					
Peak high (nmol)	*n* = 488	253.5 (201–308.1)	*n* = 460	257.9 (194.6–307.3)	0.84
ETP (nmol·min)	*n* = 488	1322 (1052–582)	*n* = 460	1304 (1063–652)	0.023
Pd‐MPs (number·mL^−1^)					
Total	*n* = 488	981.5 (518–2147)	*n* = 464	758.5 (416.5–373)	<0.0001
Annexin V+	*n* = 488	778 (409–1851)	*n* = 464	730 (380.5–412)	0.021

ETP, endogenous thrombin potential, Pd‐MPs, platelet derived microparticles.

### Risk factors for CRT

In univariate analysis, the following patient‐related covariates were significantly associated with CRT on repeated DUS as per study protocol: increased age (>50 years), obesity and presence of one or more comorbidities (Table 3). The strongest association was observed with obesity (BMI >30 kg/m²) (OR, 2.64; 95% CI, 1.46–4.76; *P* = 0.001), both in symptomatic (OR, 3.60; 95% CI, 1.22–10.6; *P* = 0.021) and asymptomatic (OR, 2.18; 95% CI, 1.11–4.26; *P* = 0.023) patients (Table 3). Patients having one or more comorbidities were at higher risk of CRT (OR, 2.05; 95% CI, 1.18–3.56; *P* = 0.011), as well as women older than 50 years at BC diagnosis (OR, 1.80; 95% CI, 1.01–3.22; *P* = 0.048). Previous history of VTE was not significantly associated with the detection of CRT (OR, 1.54, 95%CI, 0.87–2.73; *P* = 0.14). Cancer histological type was significantly associated with the risk to develop a CRT, and was lower in patients with ductal (OR, 0.55; 95% CI, 0.28–1.07; *P* = 0.078) compared to lobular carcinoma (OR, 2.53; 95% CI, 1.32–4.85; *P* = 0.005) (Table 3). CVC insertion‐related factors (such as placement site and side, more than 2 venipunctures, insertion procedure duration >20 min) were not significantly associated with CRT (Table 3), except a trend for CVC jugular insertion (OR, 1.80: 95%CI, 0.93–3.49, *P* = 0.082). There was no difference between the various original manufacturers brand of inserted CVC and the frequency nor type of VTE event. The association between each biological blood parameters and CRT occurrence was further analyzed. For Pd‐MPS and Pd‐MPs/PS+, we examined the delta between baseline and D2 values after CVC insertion while other blood parameters were analyzed as a binary variable with a threshold at the 75th percentile of the total study population as previously described 21. No statistical difference was observed between patients who developed a CRT and those who did not for any biomarkers (Table 3). Using multivariate analysis and a backward stepwise model, obesity (OR, 2.66; 95%CI, 1.46–4.84, *P* = 0.001) and lobular carcinoma histological type (OR, 2.56; 95%CI, 1.32–4.96, *P* = 0.005) remained strongly associated with the occurrence of CRT (Table 4).

**Table 3:** Catheter‐related thrombosis risk factors in univariate analysis

Variable	Symptomatic CRT 14 events in 524 pts			Asymptomatic CRT 46 events in 524 pts			All CRT 60 events in 524 pts		
	Missing on CRT+/CRT 510/14	OR	*P*	Missing on CRT+/CRT 478/46	OR	*P*	Missing on CRT+/CRT 464/60	OR	*P*
**Clinical variable**									
Age >50 years	0/0	1.31 (0.43–3.97)	0.63	0/0	1.94 (1.00–3.78)	0.052	0/0	1.80 (1.01–3.22)	0.048
BMI ≥30 kg/m^2^	0/0	3.60 (1.22–10.6)	0.021	0/0	2.18 (1.11–4.26)	0.023	0/0	2.64 (1.46–4.76)	0.001
Ductal carcinoma	0/0	0.59 (0.16–2.18)	0.43	0/0	0.56 (0.26–1.18)	0.12	0/0	0.55 (0.28–1.07)	0.078
Lobular carcinoma	0/0	2.74 (0.83–8.99)	0.097	0/0	2.28 (1.10–4.73)	0.027	0/0	2.53 (1.32–4.85)	0.005
Previous VTE	0/0	2.08 (0.71–6.11)	0.18	0/0	1.35 (0.71–2.59)	0.36	0/0	1.54 (0.87–2.73)	0.14
Presence of comorbidities	0/0	3.63 (1.24–10.7)	0.019	0/0	1.60 (0.85–3.02)	0.14	0/0	2.05 (1.18–3.56)	0.011
Subclavian CVC	0/0	0.45 (0.06–3.52)	0.45	0/0	0.55 (0.19–1.57)	0.26	0/0	0.51 (0.20–1.32)	0.17
Jugular CVC	0/0	2.60 (0.57–11.7)	0.22	0/0	1.58 (0.77–3.28)	0.22	0/0	1.80 (0.93–3.49)	0.082
Cephalic CVC	0/0	2.38 (0.29–19.3)	0.42	0/0	0.64 (0.08–4.95)	0.67	0/0	1.03 (0.23–4.63)	0.97
Right versus left CVC	23/0	1.93 (0.63–5.87)	0.25	20/3	0.76 (0.34–1.69)	0.50	20/3	1.00 (0.52–1.94)	0.99
Insertion procedure duration >20 min	126/4	3.00 (0.42–9.56)	0.39	121/9	0.79 (0.39–1.59)	0.51	117/13	0.95 (0.50–1.80)	0.87
Number of venipuncture >2	124/3	2.95 (0.61–14.4)	0.18	112/15	0.87 (0.20–3.82)	0.85	109/18	1.39 (0.46–4.21)	0.56
Distal tip of > junction SVC‐RA	99/3	0.64 (0.14–3.01)	0.57	93/9	0.79 (0.35–1.78)	0.56	90/12	0.74 (0.36–1.54	0.40
**Biological variable**									
Platelet count	11/1	1.00 (0.99–1.01)	0.60	10/2	1.00 (1.00–1.00)	0.74	9/3	1.00 (1.00–1.00)	0.58
Creatinine level	164/7	0.99 (0.94–1.04)	0.72	148/23	0.99 (0.96–1.02)	0.38	141/30	0.99 (0.96–1.01)	0.34
APPT	23/2	0.98 (0.87–1.10)	0.78	23/2	0.99 (0.96–1.03)	0.66	21/4	0.99 (0.95–1.03)	0.60
Prothrombin time	31/1	1.06 (1.00–1.12)	0.079	29/3	1.00 (0.97–1.03)	0.99	28/4	1.01 (0.98–1.05)	0.45
D‐Dimers (ng·mL^−1^) <Q3 vs. ≥Q3							
Before catheter insertion	23/0	2.43 (0.82–7.14)	0.11	29/5	1.09 (0.53–2.23)	0.82	29/5	1.39 (0.76–2.55))	0.29
After catheter insertion	23/0	1.93 (0.63–5.87)	0.25	52/7	1.35 (0.67–2.73)	0.40	50/9	1.52 (0.83–2.80)	0.18
TGT Peak (nmol) <Q3 vs. ≥Q3							
Before catheter insertion	23/0	1.21 (0.37–3.93)	0.75	20/3	1.04 (0.51–2.23)	0.92	20/3	1.08 (0.58–2.03)	0.80
After catheter insertion	23/0	1.86 (0.61–5.66)	0.27	20/3	0.99 (0.47–2.08)	0.99	20/3	1.20 (0.64–2.25)	0.57
TGT ETP (nmol·min) <Q3 vs ≥Q3							
Before catheter insertion	23/0	1.70 (0.56–5.17)	0.35	20/3	1.18 (0.59–2.38)	0.64	20/3	1.32 (0.72–2.43)	0.37
After catheter insertion	23/0	2.54 (0.86–7.48)	0.090	20/3	1.14 (0.56–2.34)	0.72	20/3	1.46 (0.79–2.69)	0.22
Pd‐MPs (number·mL^−1^) <Q3 vs ≥Q3							
Variation before‐after insertion	65/2	1.00 (0.99–1.01)	0.96	20/3	1.00 (1.00–1.00)	0.11	20/3	1.00 (1.00–1.00)	0.14
Pd‐MPs annexine V (number·mL^−1^) <Q3 vs ≥Q3							
Variation before‐after insertion	65/2	1.00 (1.00–1.00)	0.90	20/3	1.00 (1.00–1.00)	0.079	20/3	1.00 (1.00–1.00)	0.12

CRT, catheter‐related thrombosis; BMI, body mass index,; VTE, venous thromboembolism; CVC; central venous catheter; APTT, Activated Partial Thromboplastin Time; TGT, thrombin generation test; ETP, endogenous thrombin potential; Pd‐MPs, platelet derived microparticules; Q3, quartile 3; OR, odds ratio.

**Table 4:** Catheter‐related thrombosis risk factors in backward stepwise multivariate analysis

	OR	Lower	Upper	*P*‐value
Symptomatic CRT				
Comorbidities ≥1 (including diabetes mellitus, hypertension, liver failure, kidney failure, chronic respiratory failure or cardiac failure)	3.63	1.24	10.7	0.019
Asymptomatic CRT				
BMI ≥30 kg/m^2^	2.18	1.11	4.26	0.023
All CRT				
BMI ≥30 kg/m^2^	2.66	1.46	4.84	0.001
Lobular carcinoma	2.56	1.32	4.96	0.005

CRT, catheter‐related thrombosis; BMI, body mass index.

### Nested case–control study for Thrombophilia factors

None of study protocol DUS‐CRT‐patients had an antithrombin, protein S nor a protein C deficiency. There was no significant difference between patients (P) and controls (C) regarding the rates of lupus anticoagulant positivity (*P* = 0.56 for the Rosner positivity and *P* = 0.43 for DRVVT positivity), anticardiolipin antibodies positivity (*P* = 0.70), *β*2GP1 antibodies positivity (C = 1, P = 0), FVL polymorphism (C = 6; P = 1; *P* = 0.26), and prothrombin G20210A polymorphism mutation (C = 3, P = 3, *P* = 0.68). All patients and controls carrying the FVL or prothrombin G20210A polymorphisms were heterozygous.

## Discussion

Women with BC carry a three to fourfold higher risk of VTE compared with women of a similar age without cancer 2, 3, 6, 22, and important insights into time‐dependent‐related VTE risk factors during BC treatment were recently gained 6. However, previous studies investigating VTE in BC patients focused on the risk of deep vein thrombosis (DVT) and PE. They did not address the risk of CRT, while the highest VTE absolute rate in BC population was observed during the course of chemotherapy, usually administered via CVC, and within the month after cessation of chemotherapy, with, respectively, 10.8 and 8.4 cases/1000 6, 8. The use of CVC to deliver NAC in BC has considerably increased in the past decades and CRT has become a major problem in contemporary oncology practice. Indeed, CRT accounts for significant morbidity with prolonged hospitalization 23, direct increase in treatment‐related and management costs when CVC replacement is warranted 24. Therefore, it remains important to identify BC patients at higher risk for CRT.

The CAVECCAS study was specifically designed in a selected population of nonmetastatic BC patients undergoing insertion of a single lumen CVC for at least 3 months to analyze the incidence and outcomes of both asymptomatic and symptomatic CRT after CVC insertion on repeated DUS, and until 6 months of clinical follow‐up. It also aimed to a comprehensive analysis of the various clinical and biological CRT risk factors that may help clinicians to further identify BC patients who may be candidate for thromboprophylaxis.

During the 6 months of clinical follow‐up, we observed 14 symptomatic CRT (2.7%) and 46 asymptomatic CRT (8.8%) corresponding to a CRT incidence rate of 2.18 cases per 100 patient‐months. Our results are consistent with previous reports estimating the overall incidence of symptomatic CRT in the general cancer population to be lower than 5% 9. The rate of asymptomatic CRT in CAVECCAS BC patients was notably lower than previously reported in the general cancer population (between 27% and 66%) 9. The time of onset for all CRT event did not exceed 3 months after CVC insertion. These results are concordant with a large retrospective study of 5447 CVC placed for different malignancies, including 50% BC 25, where the risk of symptomatic CRT was 0.1149/1000 days catheters with 30% of CRT occurring during the first month and 48% within the first 60 days after CVC insertion. The incidence of symptomatic CRT was 0.9% at 30 days, 1.36% at 60 days, 1.83% at 90 days, and 2.22% at 120 days 25. Similarly, the median time to symptomatic CRT was 30 days among 444 consecutive patients with various cancer types prospectively followed with CVC in place for at least 4 and up to a maximum of 52 weeks 26, while earlier asymptomatic CRT (64% at 8 days and 98% at 1 month) was diagnosed on systematic phlebography in 127 of them. All together, these results indicate that the CRT risk is higher in the 3 months following CVC insertion in cancer patients.

In the CAVECCAS study, 3 out of 14 patients (21%) with symptomatic CRT presented with simultaneous nonfatal PE. In a prospective study, using systematic ventilation‐perfusion lung scan performed within 24 h of CRT diagnosis 27, PE was detected in 13/86 (15%) patients with CRT. These data underline that symptomatic CRT is a serious disease necessitating a prompt diagnosis as well as an appropriate anticoagulation 12, 14. On the contrary, none of the 46 patients with asymptomatic CRT experienced symptomatic CRT or DVT or PE up to 6 months of clinical follow‐up. A major finding of the CAVECCAS study is to point the reassuring clinical outcome of asymptomatic CRT diagnosed by DUS within the first 3 months up to 6 months of clinical follow‐up, further supporting the current international guidelines.

Much of our knowledge about CRT risk factors derived from a large meta‐analysis that included 5636 subjects with various types of unselected cancers 16. In this meta‐analysis, a previous history of DVT (OR, 2.03; 95% CI, 1.05–3.92), a subclavian venipuncture insertion (OR, 2.16; 95% CI, 1.07–4.34) and catheter tip misplacement, that is, malpositioning the tip of the CVC, which distal tip should be placed at the superior vena cava and the right atrium junction (OR, 1.92; 95% CI, 1.22–3.02) were found to increase the risk of CRT 16. In the CAVECCAS cohort, neither a history of DVT (OR, 1.54, 95%CI, 0.87–2.73; *P* = 0.14), nor the CVC insertion technique and position‐related factors were significantly associated with CRT onset. A recent Cochrane analysis concluded that jugular and subclavian CVCs insertion sites carry the same risk of CRT in long‐term recipients 28.

In CAVECCAS highly selected BC patient population, using multivariate analysis, we found that obesity (OR, 2.66: 95%CI, 1.46–4.84, *P* = 0.001) and lobular carcinoma histological type (OR, 2.56: 95%CI, 1.32–4.96, *P* = 0.005) were significantly associated with CRT. Obesity has been known as a VTE risk factor for long both in noncancer and more recently in cancer patients. Using the Khorana risk assessment scale, a BMI > 35 kg/m^2^ is an independent risk factor for subsequent DVT or PE in patients treated with chemotherapy 29 and BMI was recently shown to be a significant predictor of VTE in a large BC population (HR, 3.0; 95%CI, 2.1–4.4) 6. CAVECCAS results point out the role of obesity as a major contributor to CRT in BC patients. It first highlights the role of invasive lobular carcinoma (ILC) histological type, which has been reported to be less responsive to NAC than ductal carcinoma, probably due to differences in molecular characteristics, particularly HR and HER2 expression 30. A relationship between ILC and serum estrogen levels has been previously suggested given the increasing frequency of ILC among postmenopausal women taking hormone replacement therapy 31, 32. Interestingly, estrogens have different effects on the coagulation system resulting in a procoagulant state 33.

We also investigated if reliable biomarkers of blood coagulation activation and fibrinolysis help to identify those BC patients at higher risk of CRT and to tailor the need for CRT thromboprophylaxis. Two previous studies supported that D‐dimers levels correlate with symptomatic CRT. In a case control study by Jansen et al. 34, 30 CRT patients with a Hickman catheter undergoing allogeneic bone marrow transplantation for hematological malignancies were compared with 30 matched controls. Patients with D‐dimers levels >350 *μ*g/L measured three to 5 days after catheter insertion had a 6 times higher risk of developing subclavian CRT 34. Similar results were found in 48 patients with renal insufficiency, albeit inserted with double lumen catheters for hemodialysis 35. In CAVECCAS study, the significant increase in D‐dimers levels after catheter insertion (*P* < 0.0001), reflecting blood coagulation activation, did not predict CRT occurrence. Measures of the thrombin generation potential provide a global method to quantify the effect of the numerous genetic and environmental factors involved in the coagulation pathway. In the Vienna Cancer and Thrombosis Study (CATS), elevated thrombin peak values were associated with an increased risk of DVT or PE with a hazard ratio of 2.1 (95% CI 1.3–3.3) in multivariate analysis 21. On the contrary, in our study, neither the thrombin generation peak height nor the endogenous thrombin potential predicted CRT.

MPs have emerged as promising biomarkers due to their procoagulant properties related to the exposure of negatively charged phospholipids, mainly phosphatidyl‐serine and tissue factor. Several studies showed that increased levels of Tissue Factor positive MPs correlated with VTE or PE in cancer patients 36, but there are no data yet on the role of MPs in the occurrence of CRT in cancer patients. We therefore investigated whether Pd‐MPs were predictive of CRT in the CAVECCAS cohort. Paradoxically, we observed that both Pd‐MPs and Pd‐MPs/PS+ levels significantly decreased after CVC insertion. High levels of Pd‐MPS and Pd‐MPs/PS+ did not correlate with CRT. Our results do not support routine testing of these biomarkers in BC patients with CVC, but further studies are required to confirm these findings in patients with other malignancies.

Heritable or acquired risk factors for VTE of clinical relevance include antithrombin, protein S, protein C deficiencies, the G1691A polymorphism FVL, the prothrombin G20210A polymorphism, presence of lupus anticoagulant, positivity of anticardiolipin and anti*β*2GP1 antibodies. We performed a nested case‐control study for all these thrombophilia risk factors and found no difference between cases and matched controls for any of these. Our results appeared controversial with previous studies, which reported significant associations between FV Leiden polymorphism and CRT in acute leukemia 37, bone marrow transplant 38, unselected cancer patients 39 and in locally advanced or metastatic BC patients, all treated with the same chemotherapy protocol 40.

A limitation of our study is that it was underpowered to demonstrate the association of heritable or acquired thrombophilia with CRT occurrence. For economical and organizational reasons, it was not possible to measure antithrombin, protein C, protein S, lupus anticoagulant, anticardiolipin and anti*β*2GP1 antibodies, and to test FVL and G20210A prothrombin polymorphisms in the whole included population. Blood samples were drawn in patients with CRT and matched‐controls only and we used of a nested case control study approach.

The main strength of our study using repeated DUS is to provide current estimates of absolute risk of both asymptomatic and symptomatic CRT and their outcomes in a large sample size of 534 selected BC patients, as well as a comprehensive analysis of the multiplicity of CRT risk factors. According to current identified CRT risk factors 10, 14, 16, we chose to study a highly selected population of nonmetastatic invasive BC patients necessitating NAC via a central lumen catheter. Indeed, we aimed to investigate the specific incidence, risk factors and outcomes of both asymptomatic and symptomatic CRT in nonmetastatic BC patients within the 6 months after CVC insertion for NAC, since no study has yet analyzed this frequent clinical setting. Further studies will be necessary in BC patients with metastatic disease to analyze if the risk of CRT is higher, as already shown in metastatic cancer patients with VTE outside the context of CRT.

In summary, the CAVECCAS study provides unique clinical data on a large number of highly selected patients over a relatively long period of follow‐up. BC patients treated with NAC are at increased risk of CRT, and obesity and lobular carcinoma histological type appear as major CRT‐risk factors. Clinical parameters only allowed to identify high risk DUS‐CRT patients. Whether assessment of these risk factors may be clinically useful for an individual stratification of BC patients who might benefit from CRT prophylaxis deserves further studies.

Existing guidelines recommend not to use anticoagulation for routine prophylaxis of CRT 14, 41, Nevertheless, two studies 42, 43 and one meta‐analysis 44 suggested a potential benefit in CRT prevention using anticoagulants. In this setting, the potential benefits of new anti‐thrombotic drugs should be further evaluated.
